# Controlled Growth of an Mo_2_C—Graphene Hybrid Film as an Electrode in Self-Powered Two-Sided Mo_2_C—Graphene/Sb_2_S_0.42_Se_2.58_/TiO_2_ Photodetectors

**DOI:** 10.3390/s19051099

**Published:** 2019-03-04

**Authors:** Zhe Kang, Zhi Zheng, Helin Wei, Zhi Zhang, Xinyu Tan, Lun Xiong, Tianyou Zhai, Yihua Gao

**Affiliations:** 1 Wuhan National Laboratory for Optoelectronics (WNLO) & School of Physics & School of Materials Science and Engineering, Huazhong University of Science and Technology (HUST), Center for Nanoscale Characterization & Devices (CNCD), LuoyuRoad 1037, Wuhan 430074, China; kangzheyc@hust.edu.cn (Z.K.); zhiz890913@hust.edu.cn (Z.Z.); hlwei@hust.edu.cn (H.W.); zzhang@hust.edu.cn (Z.Z.); zhaity@hust.edu.cn (T.Z.); 2 College of Materials and Chemical Engineering, China Three Gorges University, Daxue Road 8, Yichang 443002, China; 3 Hubei Key Laboratory of Optical Information and Pattern Recognition, School of Optical Information and Energy Engineering, School of Mathematics and Physics, Wuhan Institute of Technology, Guanggu 1st Road 206, Wuhan 430205, China; xionglun@wit.edu.cn

**Keywords:** Mo_2_C, graphene, Sb_2_S_0.42_Se_2.58_, self-powered, photodetectors

## Abstract

The monotonic work function of graphene makes it difficult to meet the electrode requirements of every device with different band structures. Two-dimensional (2D) transition metal carbides (TMCs), such as carbides in MXene, are considered good candidates for electrodes as a complement to graphene. Carbides in MXene have been used to make electrodes for use in devices such as lithium batteries. However, the small lateral size and thermal instability of carbides in MXene, synthesized by the chemically etching method, limit its application in optoelectronic devices. The chemical vapor deposition (CVD) method provides a new way to obtain high-quality ultrathin TMCs without functional groups. However, the TMCs film prepared by the CVD method tends to grow vertically during the growth process, which is disadvantageous for its application in the transparent electrode. Herein, we prepared an ultrathin Mo_2_C—graphene (Mo_2_C—Gr) hybrid film by CVD to solve the above problem. The work function of Mo_2_C—Gr is between that of graphene and a pure Mo_2_C film. The Mo_2_C—Gr hybrid film was selected as a transparent hole-transporting layer to fabricate novel Mo_2_C—Gr/Sb_2_S_0.42_Se_2.58_/TiO_2_ two-sided photodetectors. The Mo_2_C—Gr/Sb_2_S_0.42_Se_2.58_/TiO_2_/fluorine-doped tin oxide (FTO) device could detect light from both the FTO side and the Mo_2_C—Gr side. The device could realize a short response time (0.084 ms) and recovery time (0.100 ms). This work is believed to provide a powerful method for preparing Mo_2_C—graphene hybrid films and reveals its potential applications in optoelectronic devices.

## 1. Introduction

The discovery of two-dimensional (2D) materials offers new possibilities for the development of electronic devices [1,2]. Electrodes are an important part of electronic devices. 2D materials represented by graphene are widely used as electrodes in optoelectronic devices because of their unique structures and unusual mechanical, electronic and optical properties [3,4,5,6]. Since different optoelectronic devices have different energy band structures, graphene cannot satisfy every optoelectronic device due to its monotonous physical properties. For example, the monotonic work function of graphene makes its heterojunction with silicon unable to achieve the highest photoelectric conversion efficiency, and it is necessary to adjust the work function of graphene by chemical doping or hybridization with other materials [7,8]. Therefore, researchers have been looking for more electrode materials as a supplement to graphene electrodes.

2D transition metal carbides (TMCs) are considered to be potential candidates for electrodes as a complement to graphene. Recently, 2D TMCs such as carbides in the MXene family (MCene) (for example, Ti_2_C_3_*T*_x_, Mo_2_C*T*_x_) were synthesized by chemically etching layered ternary transition metal-containing phases [9,10]. The MCene was described as M_n+1_C_n_*T*_x_, where M denotes transition metal and *T*_x_ stands for surface functionalization [10]. MCene-based films are considered to be good electrodes and have been used in many devices such as metal ion batteries [11], supercapacitors [12], and field effect transistors [13]. Nevertheless, although the MCene-based film obtained by spin coating has good electrical conductivity and light transmittance [14,15], the application of the film in optoelectronic devices is still limited. On the one hand, the lateral size of MCene nanosheets synthesized by the chemically etching method often ranges from 0.1 to 10 μm [9,10]. On the other hand, surface-terminating functional groups exist on the MCene surface, leading to its thermal instability [16]. Small lateral dimensions and thermal instability have made it difficult for MCene-based electrodes to meet the needs of optoelectronic devices. Fortunately, the chemical vapor deposition (CVD) method provides an effective way to prepare large-area and thermally stable TMCs [17,18,19]. However, the pure TMC film prepared by the CVD method tends to grow vertically during the growth process, which is disadvantageous for its application in the transparent electrode [20]. An increase in the thickness of TMCs results in a decrease in their light transmittance. According to reports, graphene can limit the vertical growth of TMCs during the CVD process [21]. Furthermore, hybridizing graphene with TMC is expected to obtain a transparent large-area continuous film which can be used as an electrode of photovoltaic devices.

**Figure 1:**
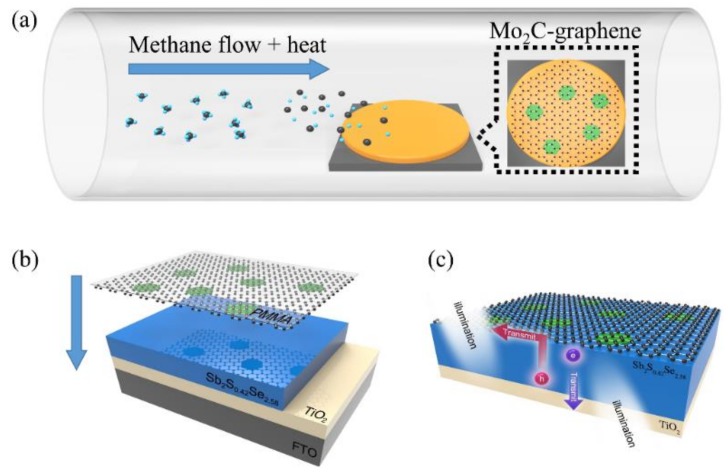
The schematic diagrams of Mo_2_C crystal growth and the novel Mo_2_C—Gr/Sb_2_S_0.42_Se_2.58_/TiO_2_/FTO vertical heterostructure photodetector. (**a**) Schematic diagram of the chemical vapor deposition (CVD) method to grown Mo_2_C—graphene. (**b**) The schematic diagram of the transfer of the Mo_2_C—Gr layer. (**c**) The schematic diagram of the self-driven two-sided photodetector.

Herein, we used the one-step CVD method to obtain a large-area ultrathin thermally stable Mo_2_C—graphene (Mo_2_C—Gr) hybrid film, as shown in Figure 1a. The work function of the Mo_2_C—Gr hybrid film was tested to be 4.07 eV. In order to acquire the high-quality Mo_2_C—Gr hybrid film, we also investigated influence factors such as CH_4_ concentration, growth time and the thickness of the Cu layer during the growth of the Mo_2_C—Gr hybrid film. Because the vertical heterostructure has been proven to be an excellent structure for photodetectors [22,23], we designed a novel Mo_2_C—Gr/Sb_2_S_0.42_Se_2.58_/TiO_2_/FTO vertical heterostructure photodetector combining the heterostructure of Mo_2_C—Gr and Sb_2_S_0.42_Se_2.58_/TiO_2_, where Sb_2_(Se_1-x_S_x_)_3_ is a promising light-absorbing material for photovoltaic device applications [24,25]. After Mo_2_C—Gr was transferred to the Sb_2_S_0.42_Se_2.58_/TiO_2_/FTO substrate, the vertical heterostructure two-sided self-powered high-speed photodetector was realized. The schematic diagram of the photodetectors is given in Figure 1b,c. The Mo_2_C—Gr/Sb_2_S_0.42_Se_2.58_/TiO_2_/FTO device could detect light from both the FTO side and the Mo_2_C—Gr side. Under 650 nm light of 2.5 mW/cm^2^ from the Mo_2_C—Gr side, the measured on/off ratio and the responsivity of the self-driven photodetector were ≈70 and 35.91 mA W^−1^, respectively. The measured voltage response and recovery time of the photodetector were 0.084 ms and 0.100 ms, respectively. We believe that our study exhibits well the application of CVD-grown 2D TMC in the optical detection. Moreover, as the abundant electrons in ultrathin TMC may be important for catalysis, this work may inspire the application of Mo_2_C—Gr in planar photocatalytic devices beyond photodetectors.

## 2. Materials and Methods

### 2.1. CVD Growth of Mo_2_C and Mo_2_C—Gr

A Cu foil (Alfa Aesar, 25 μm, 99.95% purity) was cut into 1 × 1 cm^2^ pieces and placed on the top of an Mo foil (Alfa Aesar, 50 μm, 99.95% purity) with a slightly larger size. The Cu/Mo substrate was placed in a CVD system (the outer and inner diameter of the quartz tube was 6 cm and 6.5 cm, respectively). The Cu/Mo substrates were heated to above 1090 °C under 200 sccm Ar. 0.5 sccm CH_4_ and 300 sccm H_2_ were introduced into the chamber at ambient pressure to grow the Mo_2_C crystal. 2 sccm CH_4_ and 200 sccm H_2_ were introduced into the chamber to grow the Mo_2_C—Gr.

### 2.2. Sb_2_S_0.42_Se_2.58_ Deposition and Device Fabrication

Sb_2_S_0.42_Se_2.58_/TiO_2_/FTO was obtained by rapid thermal evaporation (RTE) of Sb_2_S_0.42_Se_2.58_ on the TiO_2_/FTO substrate, like previous reports suggested [26], as shown in Figure S1. The transfer of Mo_2_C—Gr was similar to the transfer of graphene. Poly (methyl methacrylate) (PMMA) was spin-coated on the surface of Mo_2_C—Gr at 4500 r.p.m for 60 s. After PMMA cured at 170 °C for 3 min, the PMMA-coated Mo_2_C—Gr/Cu/Mo sample was cut into 0.2 × 1 cm^2^ pieces and immersed in 1 M (NH_4_)_2_S_2_O_8_ aqueous solution for etching the Cu layer. The PMMA/Mo_2_C—Gr sample was transferred to the surface of the Sb_2_S_0.42_Se_2.58_ film. PMMA was removed using warm acetone steam. Insulating tape with a window (0.2 × 1 cm^2^) adhered to the surface of the Sb_2_S_0.42_Se_2.58_ around the Mo_2_C—Gr. Ag wires were connected to the surface of Mo_2_C—Gr films with silver paint.

### 2.3. Characterizations and Measurements

An optical microscope (DM4000M, Leica, Wetzlar, Germany), scanning electron microscope (SEM) (FEI NOVA NanoSEM 450), and transmission electron microscope (TEM) (FEI Titan G2 60–300) were used to characterize the Mo_2_C crystals and Mo_2_C—Gr heterostructure. A Raman spectrum was collected by a Raman spectroscopy (LabRAM HR800, He-Ne laser excitation at 532 nm). The X-ray diffraction patterns of Mo_2_C were measured by X-ray diffraction (XRD, PANalytical B.V. X’pert PRO). A Newport 69,907 solar simulator and a Keithley 2600 SourceMeter were used for measuring the photovoltaic properties of the Mo_2_C—Gr/Sb_2_S_0.42_Se_2.58_/TiO_2_/FTO device under the condition of AM 1.5. An oscilloscope (WaveAce 1012, WaveAce, New York, NY, USA) was used to measure the response and recovery time of the device. The current-time characteristics of the photodetector were measured by a low-temperature cryogenic probe station (CRX–6.5K, Lake Shore, Westerville, OH, USA), a semiconductor parameter analyzer (4200-SCS, Keithley, Cleveland, OH, USA) and a light source (LDLS, EQ-1500, Energetiq, Woburn, MA, USA). Nyquist curves and frequency-dependent impedance were measured by the electrochemical workstation (CHI 660E, Huachen, Shanghai, China). An ultraviolet photoelectron spectroscopy (AXIS-ULTRA DLD-600W, Kratos, Tokyo, Japan) was employed for the work function measurement.

## 3. Results and Discussion

### 3.1. CVD of Mo_2_C and Mo_2_C—Graphene

**Figure 2:**
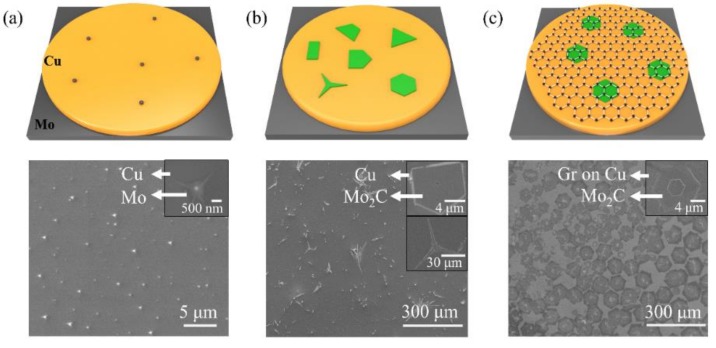
The schematic diagrams (**upper**) and SEM images (**bottom**) of Mo_2_C crystal and Mo_2_C—Gr growth under the various ratios of methane to hydrogen. (**a**) 0, (**b**) 1:600, (**c**) 1:100.

Mo_2_C crystals and Mo_2_C—Gr were synthesized by an ambient pressure CVD system. With a thermal treatment, catalyst Cu foils lying on Mo foils formed the Cu—Mo alloy as the substrate. Methane and hydrogen were pumped into the CVD system as the carbon precursor and gas reducer, respectively. At a growth temperature above 1085 °C, Cu foils melted and formed the Cu—Mo alloy at the Cu/Mo interface. The high temperature allowed Mo atoms to diffuse from the interface of Cu/Mo into the surface of liquid Cu and then formed Mo_2_C by reacting with the carbon atoms which decomposed from CH_4_. The ratio of methane to hydrogen has a great influence on the growth of Mo_2_C [27]. As shown in Figure 2a, Mo nanoparticles formed on the surface of Cu without a CH_4_ inlet. Figure 2b,c show the growth of Mo_2_C and Mo_2_C—Gr under the various ratios of methane to hydrogen, respectively. Ultrathin Mo_2_C crystals without graphene formed when the ratio was 1:600. When the ratio was 1:100, an Mo_2_C—Gr film formed on the surface of liquid Cu. The morphology of Mo_2_C crystals was greatly influenced by graphene. As shown in Figure 2b, the Mo_2_C crystals were fractal shapes at lower methane flux without graphene growth. On the contrary, Mo_2_C crystals tended to be a hexagonal shape at higher methane flux with graphene growth. As shown in Figure 2c, a hexagonal shape Mo_2_C film grew on single crystal graphene and smaller hexagonal Mo_2_C crystals often grew at the center of the film. The growth process of the vertically concentric crystals was supposed as follows. Firstly, Mo atoms diffused into the surface of liquid Cu and served as the nucleation sites for the growth of Mo_2_C and graphene. Secondly, because the growth rate of graphene (≈21 μm min^−1^) was faster than that of Mo_2_C (≈2 μm min^−1^), a large area hexagonal graphene crystal rapidly grew around the nucleation sites [21,28]. Thirdly, CH_4_ adsorbed on the grown Mo_2_C crystals, and decomposed to react with Mo atoms and form the three-layer vertically concentric Mo_2_C—Gr crystals, as shown in the inset of Figure 2c.

**Figure 3:**
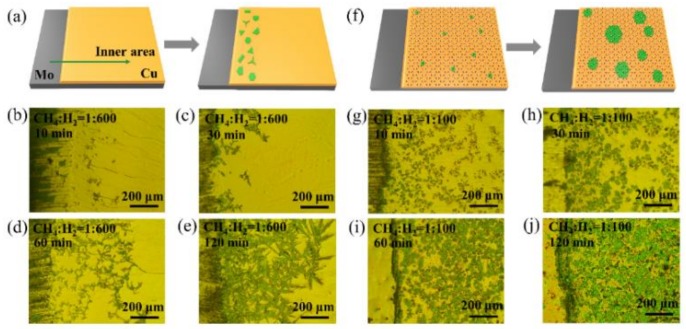
The schematic diagram and optical image showing the growth of Mo_2_C and Mo_2_C—Gr under various ratios of methane to hydrogen and various growth times. (**a**) The schematic diagram of the distribution of Mo_2_C crystals on the Cu/Mo substrate at the ratio of 1:600. (**b**–**e**) The distribution of Mo_2_C crystals on the Cu/Mo substrate with a growth time of 10, 30, 60 and 120 min, respectively. (**f**) The schematic diagram of the distribution of the grown Mo_2_C—Gr on the Cu/Mo substrate at the ratio of 1:100. (**g**–**j**) The growth of Mo_2_C—Gr and distribution of Mo_2_C crystals on the Cu/Mo substrate with a growth time of 10, 30, 60 and 120 min, respectively.

Graphene had a great influence not only on the morphology of Mo_2_C but also the macroscopic distribution of Mo_2_C crystals on the surface of liquid Cu. Figure 3a shows the schematic diagram of the distribution of grown Mo_2_C on Cu without the assistance of the graphene layer. As shown in the optical images in Figure 3b–e, most Mo_2_C grew at the edge area of the Cu substrate at the ratio of 1:600 of methane to hydrogen. Mo_2_C rarely grew in the inner area of the liquid Cu surface. This phenomenon may be caused by the surface tension of liquid Cu leading to a thinner thickness of the liquid Cu at the edge area than that in the inner area. Therefore, Mo atoms first diffused to the Cu surface of the edge area and formed Mo_2_C crystals. Mo_2_C crystals grew from the edge to the center of the Cu substrate with time increasing. However, Mo_2_C was still far from covering the Cu surface at the growth time of 120 min. As shown in Figure 3f, with the growth of the graphene layer, Mo_2_C nucleation sites uniformly distributed on the Cu substrate and then grew as large Mo_2_C crystals. This result was caused by the diffusion of C obtained by methane cracking, which only got through the grain boundary and/or defect of graphene to act with Mo atoms. Figure 3g–j show that the Mo_2_C crystals grew at various growth times. Mo_2_C almost covered the Cu surface with a 120 min growth time at the ratio of 1:100 of methane to hydrogen. Although the Mo_2_C crystals tended to become thicker under the growth condition of the high ratio, the thickness of Mo_2_C crystals could be controlled by adjusting the thickness of the Cu substrate. Under the same condition of growth, the Mo_2_C was thinner as the layer of Cu foil increased, as shown in Figure S2a–f. So far, we obtained the large area ultrathin Mo_2_C—Gr.

**Figure 4:**
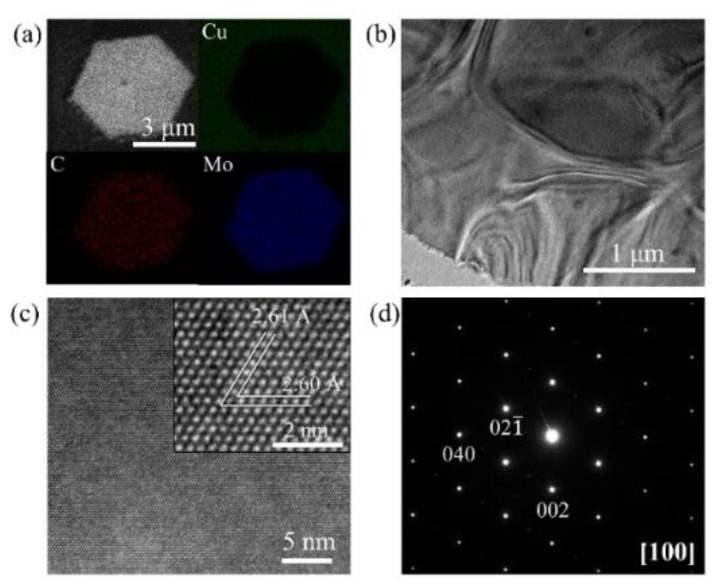
The characterization analysis of Mo_2_C. (**a**) The element distribution of Mo_2_C on Cu. (**b**) The transmission electron microscope (TEM) image. (**c**) The high-resolution TEM image of Mo_2_C. The inset image is a magnified image of a selected region. (**d**) The selected area electron diffraction (SAED) pattern along the [100] zone axis.

The element distribution and proportions of the Mo_2_C crystal were investigated by an energy dispersive spectrometer (EDS) analysis, as shown in Figure 4a and Figure S3. The proportion of Mo and C was approximately 2:1. Figure 4b shows the TEM image of Mo_2_C on microgrids. The Mo_2_C wrinkles with the surface topography of microgrids indicated that the Mo_2_C thin film had enough flexibility to contact with the substrate well. Figure 4c,d show the high-resolution transmission electron microscope (HRTEM) image and selective area electron diffraction (SAED) pattern along the [100] zone axis of Mo_2_C, respectively. The interplanar distances for the ($02\bar{1}$) and (002) planes were 2.61 Å and 2.60 Å, respectively. These interplanar distances were consistent with those of the orthorhombic α-Mo_2_C [29].

**Figure 5:**
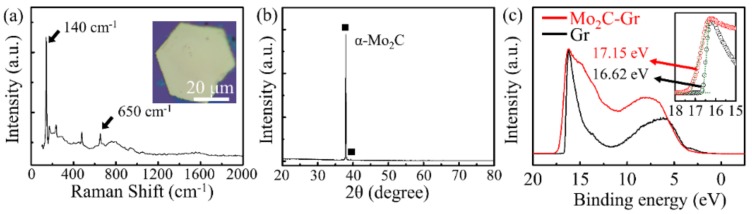
The spectral analysis of Mo2C and Mo_2_C—Gr. (**a**) The Raman spectrum of Mo_2_C. The inset is the optical image of Mo_2_C. (**b**) The X-ray diffraction patterns of Mo_2_C. (**c**) The UPS spectra of graphene (**black**) and Mo_2_C—Gr (**red**) on n-type silicon. The inset of the left panel shows the magnified region from 18 eV to 15 eV.

The optical image of Mo_2_C—Gr on the SiO_2_ substrate and the Raman spectrum of graphene are shown in Figure S4a,b, respectively. The optical image clearly shows that graphene connected the Mo_2_C crystals well. The graphene film was identified as having a few layers, as demonstrated by the ratio 1.7 of 2D peak to G peak in the Raman spectrum of graphene. As shown in Figure 5a, two characteristic peaks of the Raman spectrum of Mo_2_C crystals were near 140 cm^−1^ and 650 cm^−1^, respectively. This result matches well with previous reports [20,30]. The X-Ray Diffraction (XRD) spectrum of Mo_2_C, as shown in Figure 5b, indicated that Mo_2_C by the CVD method was the α phase. The work function was an important parameter for the electrode material. Electrodes with different work functions are suitable for use in different semiconductor devices. To gain insight into the electronic structures of Mo_2_C—Gr, the work function of graphene (tested on n-type silicon) and Mo_2_C—Gr (tested on n-type silicon) were investigated by ultraviolet photoelectron spectroscopy (UPS), as shown in Figure 5c. The work function of materials can be calculated by subtracting the secondary electron cut-off energy from the incident ultraviolet photon energy [25]. The photon energy of exciting radiation was 21.22 eV and the secondary electron cut-off energy of graphene and Mo2C—Gr was 16.62 eV and 17.15 eV, respectively. The work function of graphene and Mo_2_C—Gr on n-Si was calculated to be 4.60 eV and 4.07 eV, respectively. This result indicated that the work function of Mo_2_C—Gr can be adjusted by controlling the content of Mo_2_C. This makes it possible to design electrode materials, of different work functions, between 3.8 eV (the work function of Mo_2_C) and 4.6 eV (the work function of graphene) [31,32,33], according to the requirements of different energy band structure devices.

### 3.2. Mo_2_C—Gr/Sb_2_S_0.42_Se_2.58_/TiO_2_ Photodetectors

**Figure 6:**
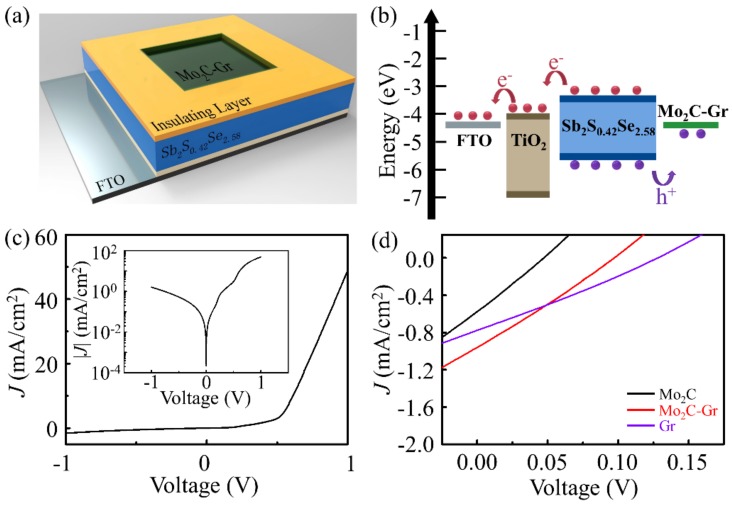
The optoelectronic characteristics of the Mo_2_C—Gr/Sb_2_S_0.42_Se_2.58_/TiO_2_ photodetector. (**a**) The schematic diagram of photodetectors. (**b**) The energy band diagram of Mo_2_C—Gr, Sb_2_S_0.42_Se_2.58_, TiO_2_ and FTO in the photodetector. (**c**) Dark current-voltage of Mo_2_C—Gr/Sb_2_S_0.42_Se_2.58_/TiO_2_ photodetectors. (**d**) Current-voltage curves of Mo_2_C/Sb_2_S_0.42_Se_2.58_/TiO_2_, Mo_2_C—Gr/Sb_2_S_0.42_Se_2.58_/TiO_2_ and Gr/Sb_2_S_0.42_Se_2.58_/TiO_2_ photodetectors, respectively, under 1.5 G illumination (100 mW/cm^−2^).

Considering that the ultrathin TMC—graphene has outstanding electrical conductivities, adjustable work function and good transparency, we used Mo_2_C—Gr as the transparent electrode and hole collector to fabricate Mo_2_C—Gr/Sb_2_S_0.42_Se_2.58_/TiO_2_ two-sided photodetectors. The schematic diagram and the energy band diagram of the Mo_2_C—Gr/Sb_2_S_0.42_Se_2.58_/TiO_2_ device are shown in Figure 6a and b, respectively. The schematic diagram of the fabrication process of the devices is given in Figure S1. EDS of Sb_2_S_0.42_Se_2.58_ is shown in Figure S5. Sb_2_S_0.42_Se_2.58_ worked as the light absorber in this configuration. Upon illumination, the photogenerated electron—hole pair was generated in Sb_2_S_0.42_Se_2.58_, and was then transported into the planar TiO_2_ and Mo_2_C—Gr layer, respectively. Figure 6c shows the dark current-voltage characteristics of the Mo_2_C—Gr/Sb_2_S_0.42_Se_2.58_/TiO_2_ device. The low dark current in the device suggests a good contact and tiny carrier recombination at the interface of the device [5]. In contrast to the one-sided photodetector which uses the Au electrode [26], the Mo_2_C—Gr/Sb_2_S_0.42_Se_2.58_/TiO_2_/FTO device, as a two-sided photodetector, can detect the light irradiating both from the Mo_2_C—Gr side and the FTO side. The current-voltage characteristics of the Mo_2_C—Gr/Sb_2_S_0.42_Se_2.58_/TiO_2_/FTO photodetector are shown in Figure S6a. The photodetector had a better photoelectric response from the FTO side than that from the Mo_2_C—Gr side, under illumination. The response difference may have originated from different absorption of light at both sides because of different transmittance of the materials at both sides. Figure 6d shows that the Mo_2_C—Gr/Sb_2_S_0.42_Se_2.58_/TiO_2_ device had a larger short-circuit current and open-circuit voltage than that of Mo_2_C/Sb_2_S_0.42_Se_2.58_/TiO_2_ devices. The reason for the bad performance of the Mo_2_C/Sb_2_S_0.42_Se_2.58_/TiO_2_ devices may be that the pure Mo_2_C film grown by CVD was too thick to transmit light. The Mo_2_C—Gr/Sb_2_S_0.42_Se_2.58_/TiO_2_ device had a larger short-circuit current but a smaller open-circuit voltage than that of the Gr/Sb_2_S_0.42_Se_2.58_/TiO_2_ device. The response difference may have originated from the fact that Mo_2_C—Gr had better conductivity than pure Gr but blocked more incident light. Considering that self-powered photodetectors typically use current response at zero bias as the output signal, the performance of the Mo_2_C—Gr/Sb_2_S_0.42_Se_2.58_/TiO_2_ photodetectors was more advantageous. The Mo_2_C—Gr/Sb_2_S_0.42_Se_2.58_/TiO_2_ photodetector had a lager current response than that of the Mo_2_C/Sb_2_S_0.42_Se_2.58_/TiO_2_ and Gr/Sb_2_S_0.42_Se_2.58_/TiO_2_ photodetector, as shown in Figure S6b.

**Figure 7:**
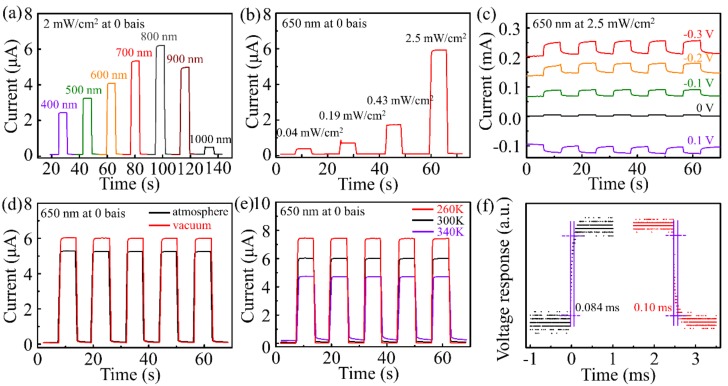
The detection performance of the Mo_2_C—Gr/Sb_2_S_0.42_Se_2.58_/TiO_2_/FTO photodetector under illumination from the Mo_2_C—Gr side. (**a**) The current-time curves of the photodetector under a 2 mW/cm^2^ illumination with a wavelength of 400 nm to 1000 nm at 0 bias. (**b**) Self-powered photoresponse under an illumination of 650 nm light. (**c**) At various bias voltages, the photoresponse under a 2.5 mW/cm^2^ illumination with 650 nm wavelength. (**d**) The current-time curves of the photodetector in atmosphere and vacuum under a 2 mW/cm^2^ illumination of 650 nm wavelength light at 0 bias. (**e**) Photoresponse at various temperatures. (**f**) The voltage response and recovery time of the photodetector.

Figure 7 shows the detection performance of the Mo_2_C—Gr/Sb_2_S_0.42_Se_2.58_/TiO_2_/FTO photodetector under illumination from the Mo_2_C—Gr side. Figure 7a shows the self-powered current-time curves of the photodetector under a 2 mW/cm^2^ irradiation of 400 nm to 1000 nm wavelength. The photocurrent response increase under illumination of 400 nm to 800 nm wavelength and then rapidly descended when the wavelength increased to 1000 nm. As shown in Figure 7b, the photoresponse increased as the light density increased. The on/off ratio (*R*_on/off_) and the responsivity (*R*_A_) of the Mo_2_C—Gr/Sb_2_S_0.42_Se_2.58_/TiO_2_/FTO photodetector were measured to be ≈70 and 35.91 mA W^−1^ under 650 nm illumination of 2.5 mW/cm^2^ without bias voltage, respectively. The Mo_2_C—Gr/Sb_2_S_0.42_Se_2.58_/TiO_2_/FTO device can be stably operated at a bias voltage, as shown in Figure 7c. The photocurrent responses and dark current responses increased with the increase of bias voltage. This phenomenon was helpful for the Mo_2_C—Gr/Sb_2_S_0.42_Se_2.58_/TiO_2_/FTO device in other applications such as electro-catalysis and the photoelectric catalysis field. The performance of the photodetector was also influenced by the atmosphere as shown in Figure 7d. The photocurrent increased and the dark current slightly declined with the improvement in vacuum degree. Figure 7e shows the influence of temperature on the performance of Mo_2_C—Gr/Sb_2_S_0.42_Se_2.58_/TiO_2_/FTO photodetectors. The photocurrent increased and the dark current decreased as the temperature dropped. As shown in Figure 7f, the response and recovery time of the Mo_2_C—Gr/Sb_2_S_0.42_Se_2.58_/TiO_2_/FTO photodetector were measured as 0.084 ms and 0.100 ms, respectively, which was much shorter than that of graphene or reduced graphene oxide on a semiconductor photodetector [34].

**Figure 8:**
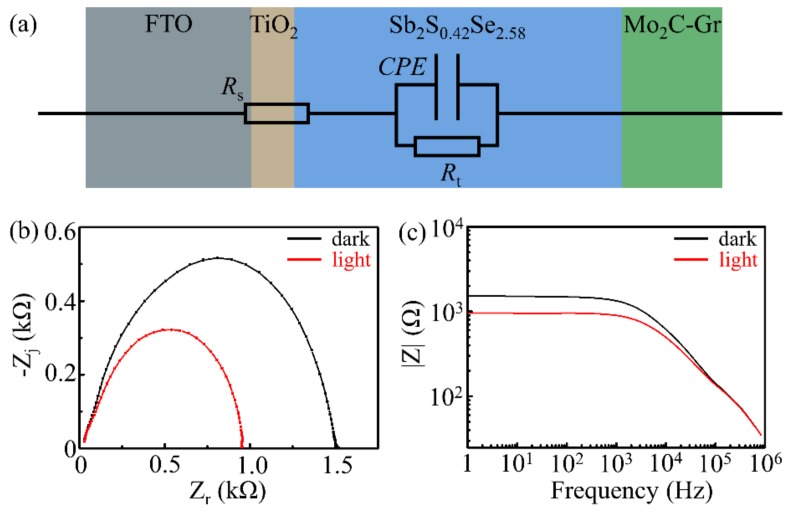
The impedance analysis of the photodetector. (**a**) The schematic equivalent circuit diagram of the Mo_2_C—Gr/Sb_2_S_0.42_Se_2.58_/TiO_2_/FTO photodetector. (**b**,**c**) Nyquist diagram and frequency-dependent relationships of the Mo_2_C—Gr/Sb_2_S_0.42_Se_2.58_/TiO_2_/FTO photodetector in the dark (**black**) and under illumination (**red**), respectively. CPE: constant-phase element.

To further elucidate the charge transportation process, electrochemical impedance spectroscopic (EIS) measurements were conducted under illumination on/off conditions without bias voltage. Figure 8a shows the equivalent circuit diagram for a Mo_2_C—Gr/Sb_2_S_0.42_Se_2.58_/TiO_2_/FTO photodetector, where *R*_s_ represents interfacial series resistances. The constant-phase element (CPE) denoted interfacial capacitances, where *R*_t_ stands for the charge-transfer resistance of the device [35,36,37]. Nyquist curves and frequency-dependent impedance is shown in Figure 8b,c. No matter the conditions, i.e., dark or illumination, as shown in Figure S7, the fitted data utilizing models invoking CPE matched well with the measured data. The value of *R*_s_ and *R*_t_ under illumination was 21.34 Ω and 966.7 Ω, respectively. The characteristic frequencies of Mo_2_C—Gr/Sb_2_S_0.42_Se_2.58_/TiO_2_/FTO devices were approximately 7 kHz. This means that the Sb_2_S_0.42_Se_2.58_ film had a few defects [26].

## 4. Conclusions

In conclusion, the CVD method proved an easy way of synthesizing the Mo_2_C—Gr hybrid film. At a high ratio of methane to hydrogen, Mo_2_C—Gr was obtained on the Cu/Mo substrate. On the one hand, graphene worked as a blocking layer during the growth of Mo_2_C to make the ultrathin Mo_2_C crystals grow uniformly and regularly. On the other hand, graphene worked as the connector between ultrathin Mo_2_C crystals. Mo_2_C—Gr, which had a work function between that of graphene and that of pure Mo_2_C, was a potential candidate for electrodes as a complement to graphene. The Mo_2_C—Gr hybrid film was used for fabricating the Mo_2_C—Gr/Sb_2_S_0.42_Se_2.58_/TiO_2_/FTO vertical structure two-sided photodetector. This photodetector showed high performance at different temperatures, bias voltages, wavelengths and intensities of incident light. The voltage response and recovery time were 0.084 ms and 0.100 ms, respectively. The responsivity of the self-powered photodetector was 35.91 mA W^−1^ under 650 nm illumination of 2.5 mW/cm^2^. We believe that our research exhibits an application of a CVD-grown Mo_2_C—Gr hybrid film in the optical detection well. Moreover, considering the abundant electrons of ultrathin TMC, important for catalysis, we believe that this work may inspire the application of the Mo_2_C—Gr hybrid film in planar photocatalytic devices beyond photodetectors.

## Supplementary Materials

The following are available online at https://www.mdpi.com/1424-8220/19/5/1099/s1. Figure S1: The preparation process of the Mo2C—Gr/Sb2S0.42Se2.58/TiO2/FTO photodetector. Figure S2: The thickness of Mo2C crystals grown on various amounts of thickness of the Cu layer: (a) 25 μm, (b) and (c) 125 μm, (d) to (f) 250 μm. Figure S3: The energy dispersive spectrometer (EDS) spectra and the atomic ratio of Mo2C. Figure S4: Characterization and analysis of graphene in the Mo2C—Graphene Structure: (a) The optical image of Mo2C—Gr. (b) The Raman spectra of graphene. Figure S5: The energy dispersive spectrometer (EDS) spectra and the atomic ratio of Sb2S0.42Se2.58/TiO2/FTO. Figure S6: (a) Current-voltage curves of the Mo2C—Gr/Sb2S0.42Se2.58/TiO2 photodetector under 1.5 G illumination (100 mW cm−2) from the Mo2C—Gr (red) side and FTO side (black). (b) Current response of Mo2C/Sb2S0.42Se2.58/TiO2, Mo2C—Gr/Sb2S0.42Se2.58/TiO2 and Gr/Sb2S0.42Se2.58/TiO2 photodetectors, respectively, under 1.5 G illumination. Figure S7: Impedance analysis of the photodetector: (a) Nyquist diagram (black) and fitted curve (red) of the Mo2C—Gr/Sb2S0.42Se2.58/TiO2/FTO photodetector under the dark condition. (b) Nyquist diagram (black) and fitted curve (red) of the Mo2C—Gr/Sb2S0.42Se2.58/TiO2/FTO photodetector under the illumination condition.
