# Supplementary material for: Controlled Growth of an Mo2C—Graphene Hybrid Film as an Electrode in Self-Powered Two-Sided Mo2C—Graphene/Sb2S0.42Se2.58/TiO2 Photodetectors
Source: Sensors (Basel). 2019 Mar 4;19(5):1099. doi: 10.3390/s19051099 (PMC6427578; doi:10.3390/s19051099)
Supplement: Supplementary file 1 [file sensors-19-01099-s001.pdf]

Supporting Information

Controlled Growth of an Mo_2C—Graphene hybrid film as an Electrode in Self-Powered Two-Sided Mo_2C—Graphene/$Sb_2S_{0.42}Se_{2.58}$/TiO_2 Photodetectors

Zhe Kang[a], Zhi Zheng[a], Helin Wei[a], Zhi Zhang[a], Xinyu Tan[b]*, Lun Xiong[c], Tianyou Zhai[a] and Yihua Gao[a,c]*

[a] Center for Nanoscale Characterization & Devices (CNCD), Wuhan National Laboratory for Optoelectronics (WNLO) & School of Physics & School of Materials Science and Engineering, Huazhong University of Science and Technology (HUST), LuoyuRoad 1037, Wuhan 430074, China.

[b] College of Materials and Chemical Engineering, China Three Gorges University, Daxue Road 8, Yichang 443002, China.

[c] Hubei Key Laboratory of Optical Information and Pattern Recognition, School of Optical Information and Energy Engineering, School of Mathematics and Physics, Wuhan Institute of Technology, Guanggu 1st Road 206, Wuhan 430205, China.

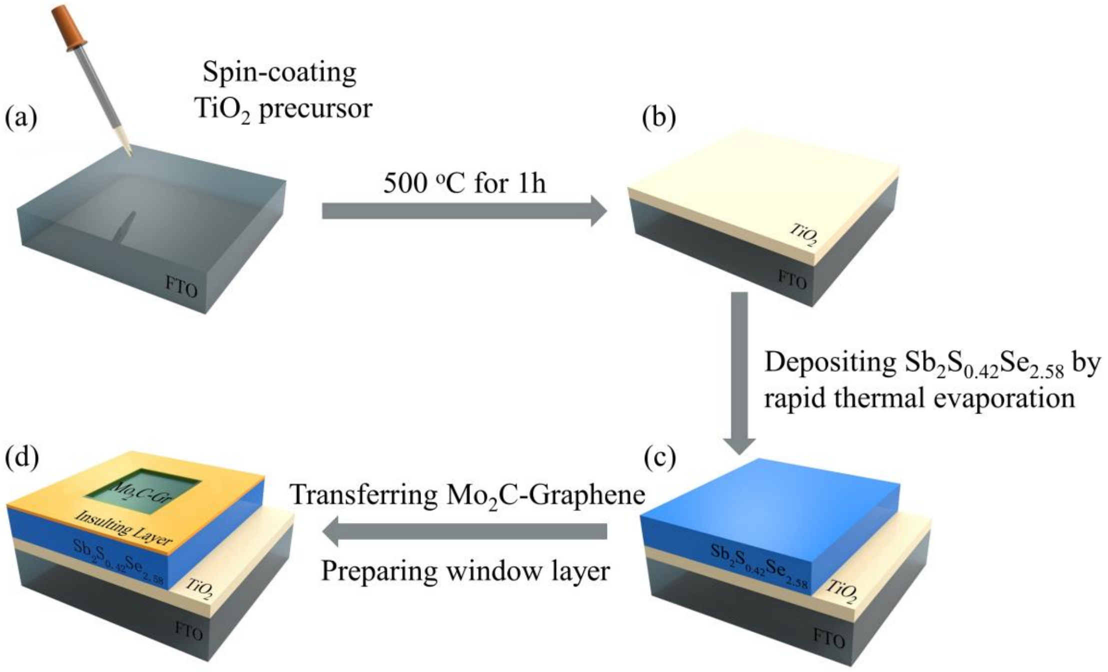

Figure S1. The preparation process of the $Mo_2CGr/Sb_2S_{0.42}Se_{2.58}/TiO_2$/FTO photodetector.

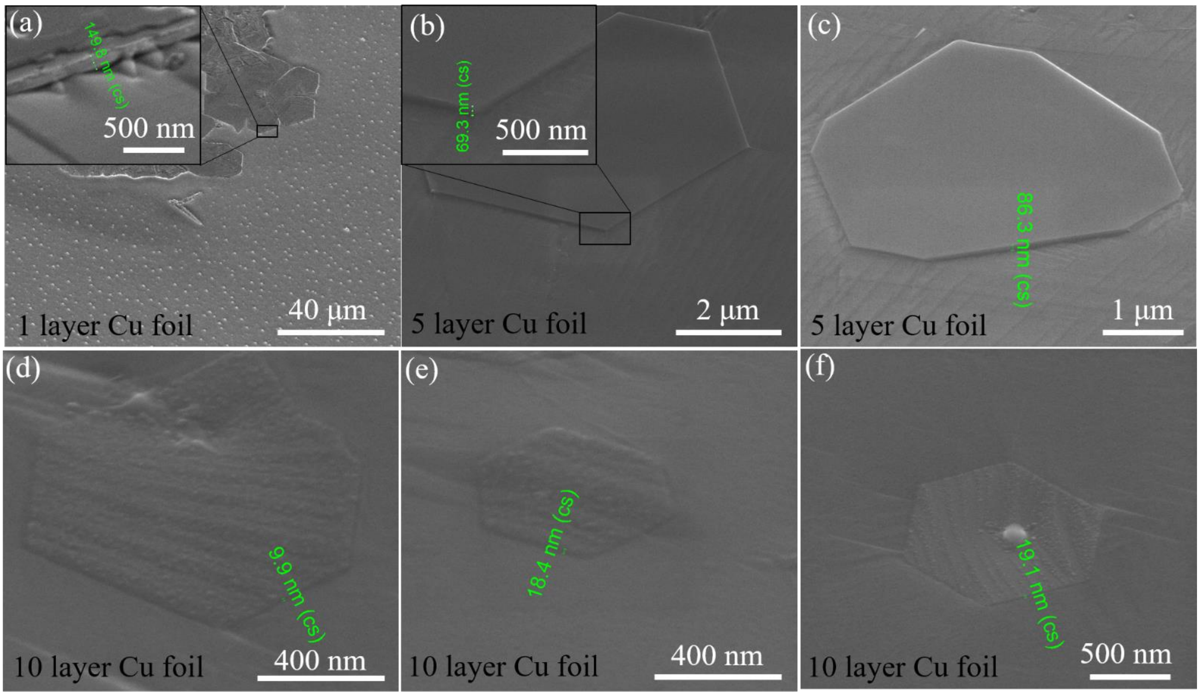

Figure S2. The thickness of Mo_2C crystals grown on various amounts of thickness of the Cu layer: (**a**) 25 μm, (**b**) and (**c**) 125 μm, (**d**) to (**f**) 250 μm.

kV : 200.00 Azimuth: 0.00 Elevation:35.00 AmpT : 102.4
Detector Type:SUTW, Sapphire Resolution:138.23 Lsec:8

Element	Weight %	Atomic %
C K	5.7	32.7
MoK	94.3	67.3
Total	100.0	100.0

Element	Net Inte.	Bkgd Inte.	Inte. Error	P/B
C K	21.36	16.14	11.81	1.32
MoK	236.74	13.65	2.36	17.35

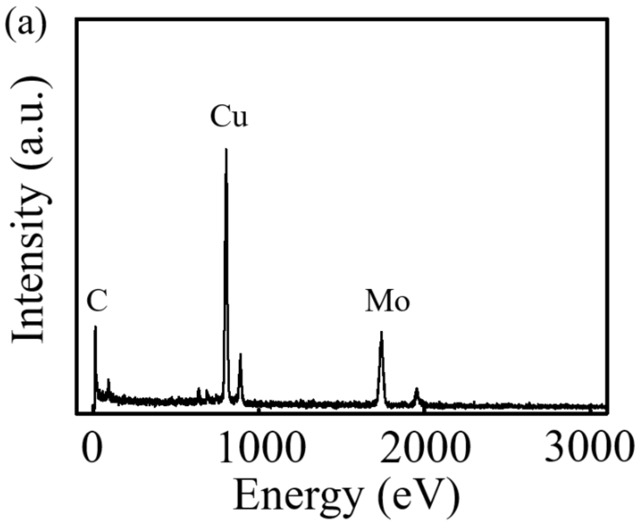

Figure S3. The energy dispersive spectrometer (EDS) spectra and atomic ratio of Mo_2C.

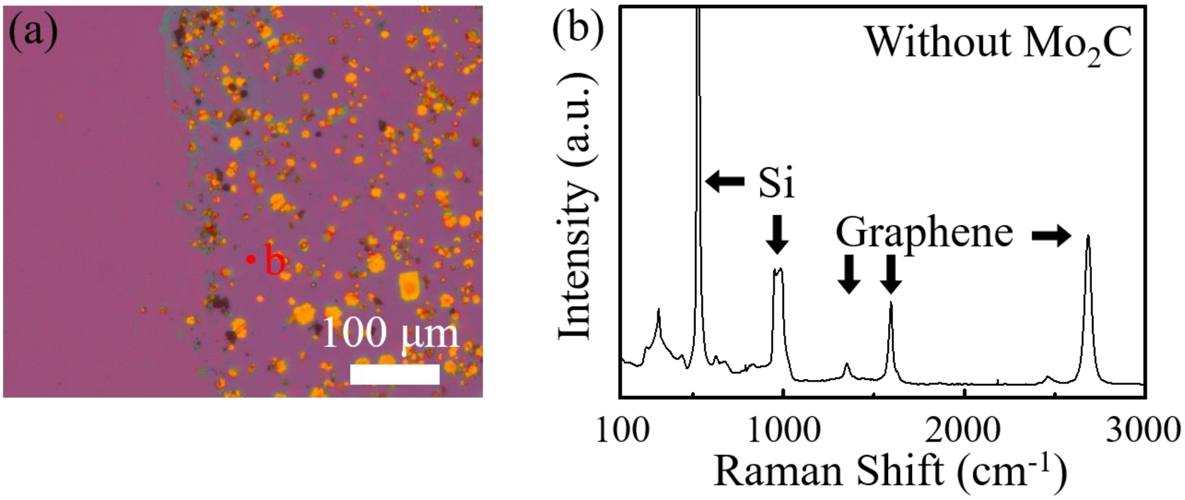

Figure S4. Characterization and analysis of graphene in the Mo_2C—graphene Structure: (**a**) The optical image of Mo_2C—Gr. (**b**) The Raman spectra of graphene.

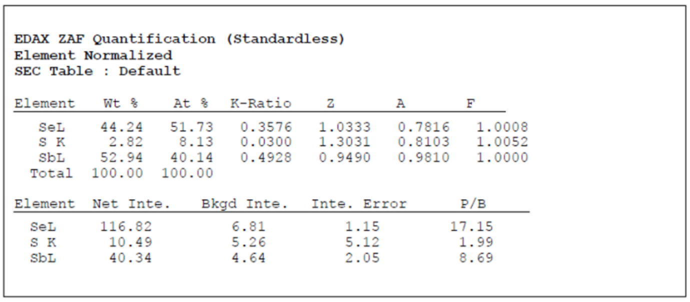
EDAX ZAF Quantification (Standardless)
Element Normalized
SEC Table : Default

Element	Wt %	At %	K-Ratio	Z	A	F
SeL	44.24	51.73	0.3576	1.0333	0.7816	1.0008
S K	2.82	8.13	0.0300	1.3031	0.8103	1.0052
SbL	52.94	40.14	0.4928	0.9490	0.9810	1.0000
Total	100.00	100.00				

Element	Net Inte.	Bkgd Inte.	Inte. Error	P/B
SeL	116.82	6.81	1.15	17.15
S K	10.49	5.26	5.12	1.99
SbL	40.34	4.64	2.05	8.69

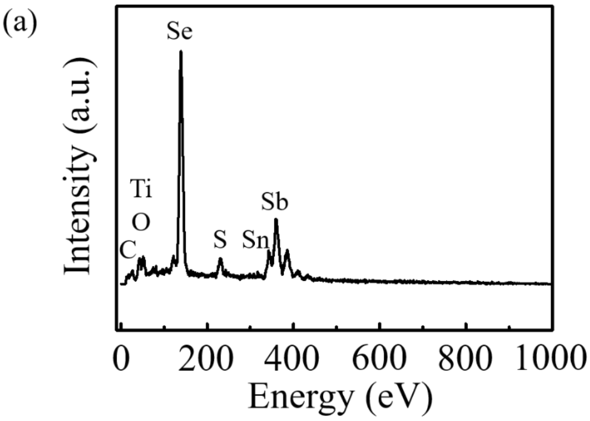

Figure S5. The energy dispersive spectrometer (EDS) spectra and atomic ratio of $Sb_2S_{0.42}Se_{2.58}/TiO_2$/FTO.

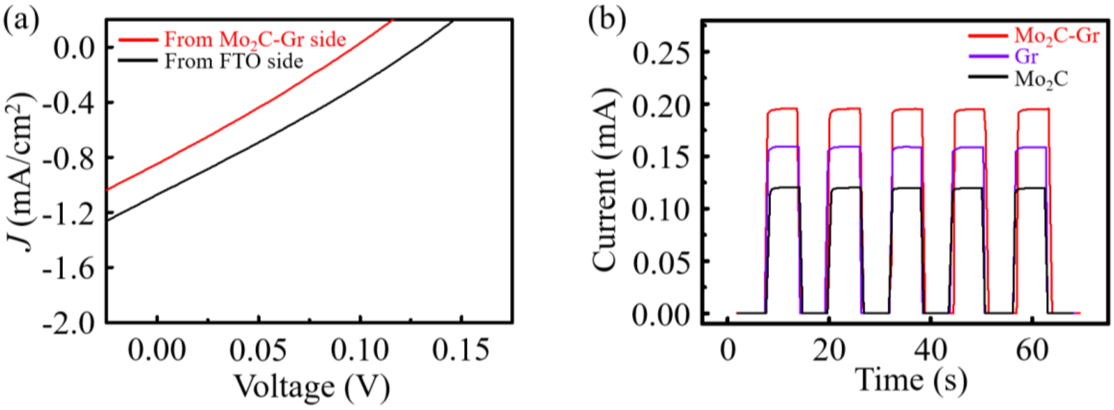

Figure S6. (**a**) Current-voltage curves of the Mo_2C-Gr/$Sb_2S_{0.42}Se_{2.58}/TiO_2$ photodetector under 1.5 G illumination (100 mW cm^{-2}) from the Mo_2C—Gr (red) side and FTO side

(black). (**b**) Current response of $Mo_2C/Sb_2S_{0.42}Se_{2.58}/TiO_2$, Mo_2C-$Gr/Sb_2S_{0.42}Se_{2.58}/TiO_2$ and $Gr/Sb_2S_{0.42}Se_{2.58}/TiO_2$ photodetectors, respectively, under 1.5 G illumination.

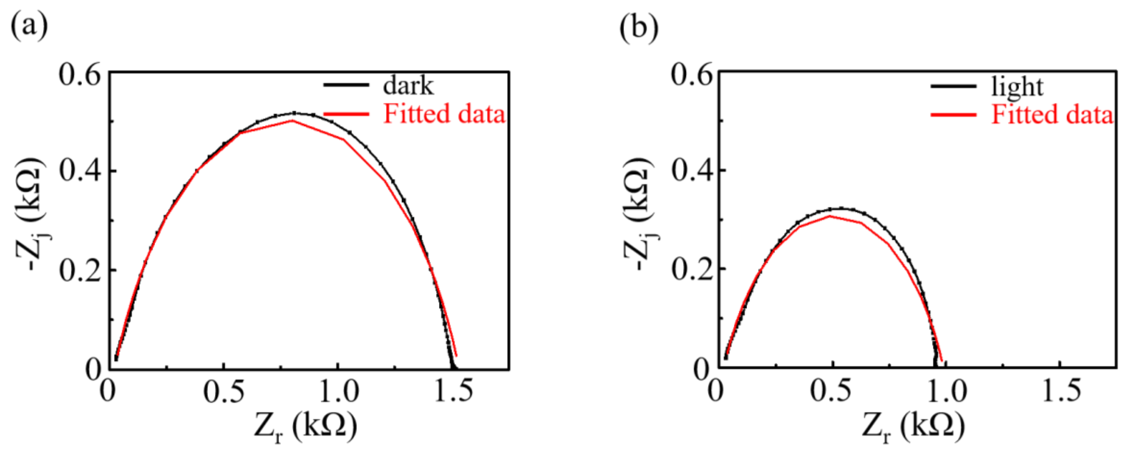

Figure S7. Impedance analysis of the photodetector. (**a**) Nyquist diagram (black) and fitted curve (red) of the Mo_2C-$Gr/Sb_2S_{0.42}Se_{2.58}/TiO_2$/FTO photodetector under the dark condition. (**b**) Nyquist diagram (black) and fitted curve (red) of the Mo_2C-$Gr/Sb_2S_{0.42}Se_{2.58}/TiO_2$/FTO photodetector under the illumination condition.
